# Supplementary material for: Auxin-dependent control of a plasmodesmal regulator creates a negative feedback loop modulating lateral root emergence
Source: Nat Commun. 2020 Jan 17;11:364. doi: 10.1038/s41467-019-14226-7 (PMC6969147; doi:10.1038/s41467-019-14226-7)
Supplement: Supplementary file 1 — Supplementary Information [file 41467_2019_14226_MOESM1_ESM.pdf]

Supplementary Figure 1

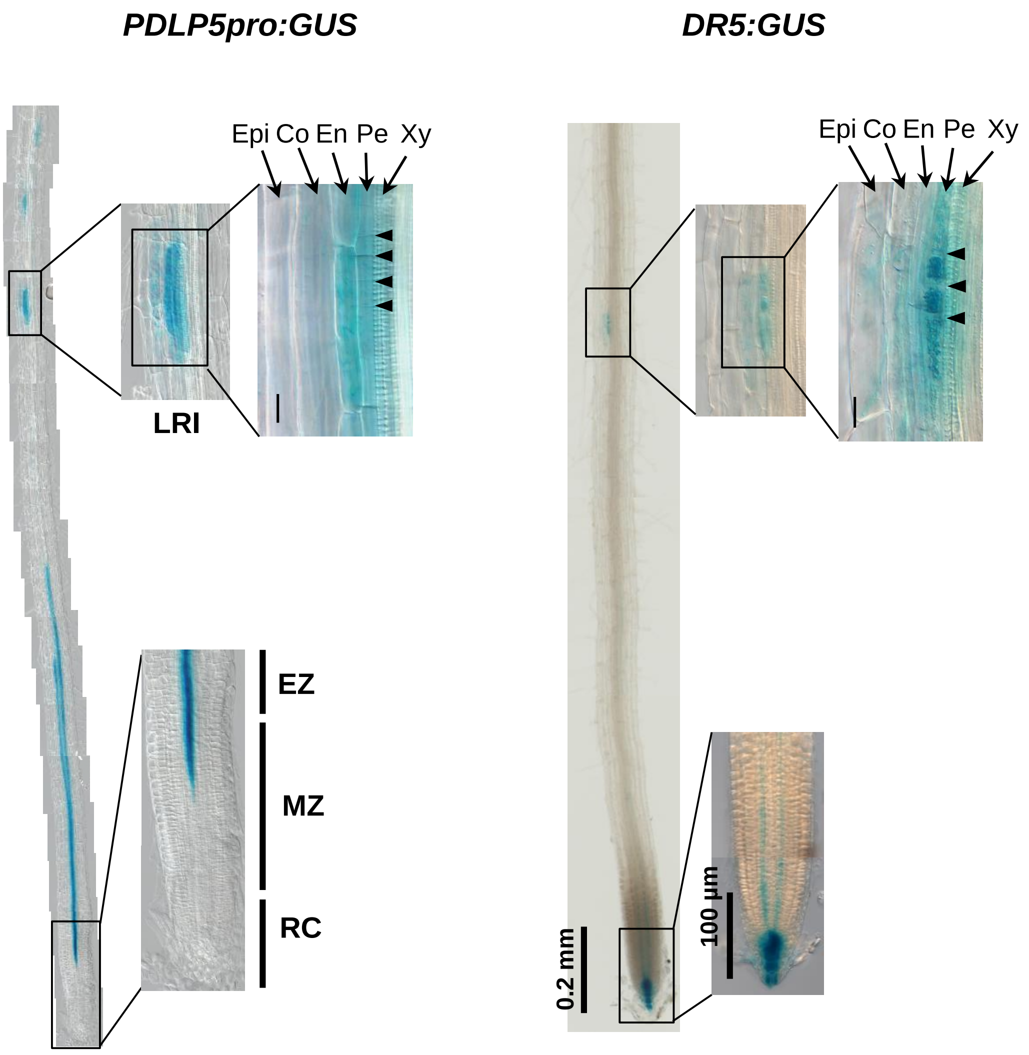

Supplementary Figure 1: *PDLP5pro:GUS* expression in protoxylem and LRP-overlying cells. Seven-day-old seedlings, GUS-stained for 2 hrs. LRI, lateral root initiation site; EZ, elongation zone; MZ, meristematic zone; RC, root cap. Darts, cell walls of newly initiating LRP.

Supplementary Figure 2

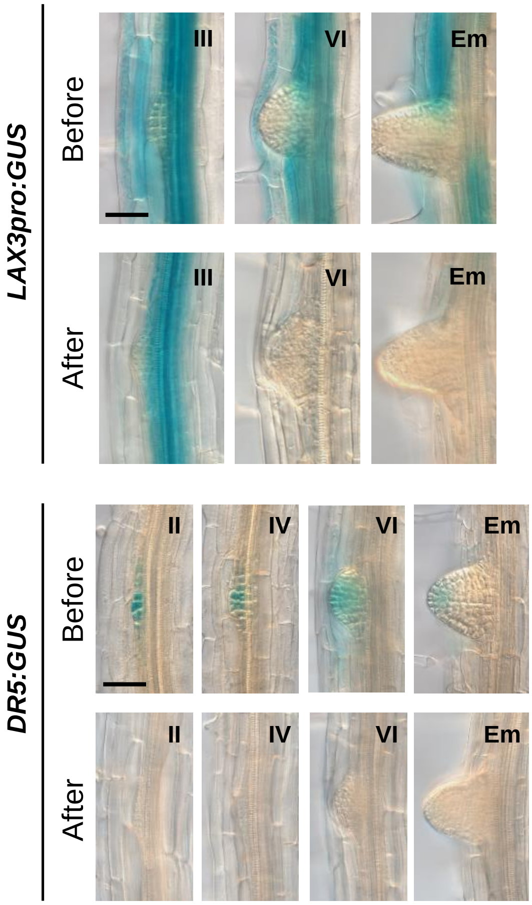

Supplementary Figure 2: *LAX3pro:GUS* and *DR5:GUS* expression in roots after shoot removal. GUS-stained 7-day-old seedlings before and after shoot removal. Shoots were removed at 5 days post germination. Roots were stained two days after shoot removal. Scale bar, 50 µm; common to all panels.

Supplementary Figure 3

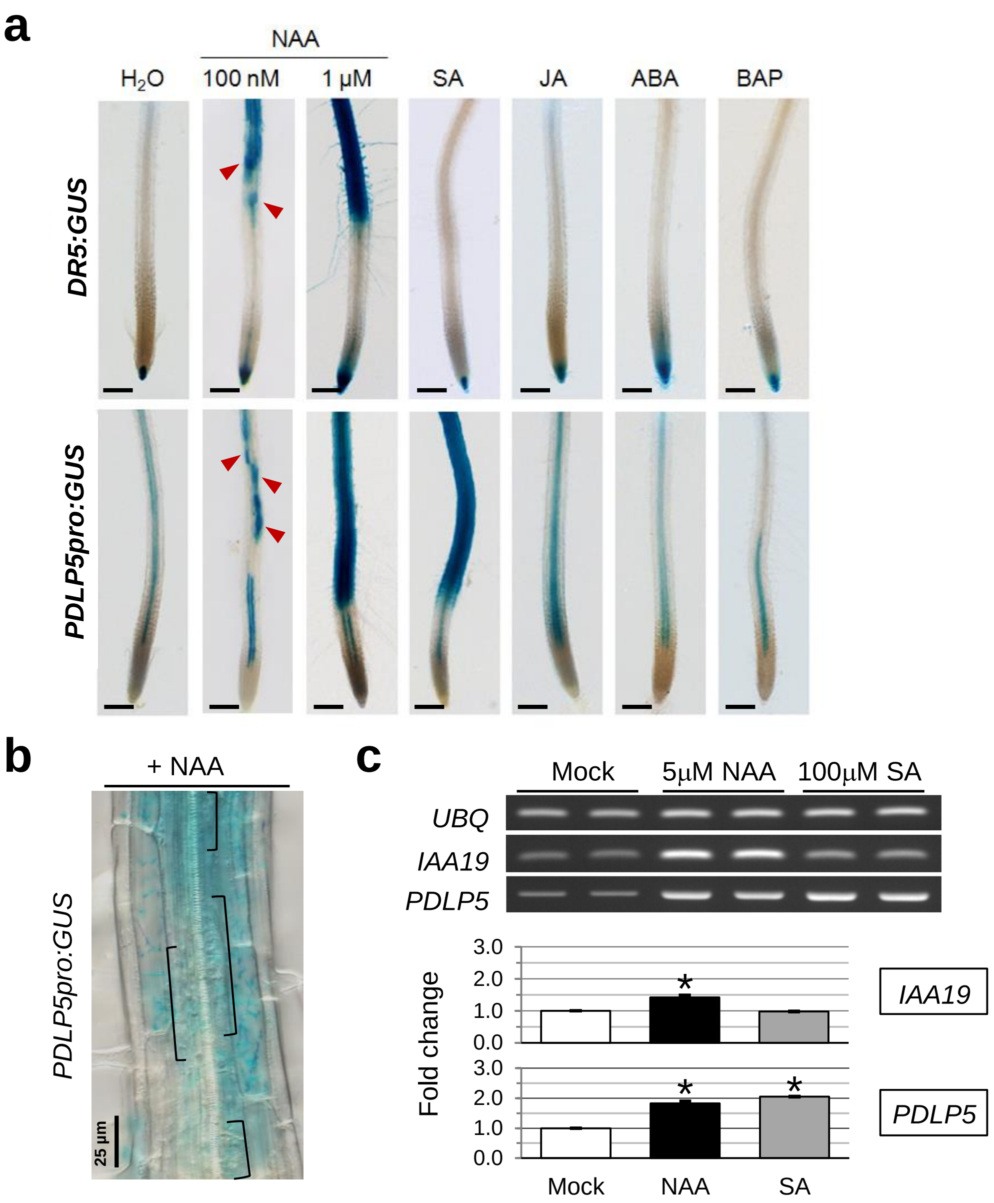

Supplementary Figure 3: Hormonal treatment of *PDLP5pro:GUS* in roots. (**a**) Seven-day-old *PDLP5pro:GUS* or *DR5:GUS* seedlings grown on MS plates were mock-treated with water drops or treated with the indicated hormones in drops. Nine hours post treatment, the seedlings were subjected to GUS staining for 3 hrs. Concentrations: salicylic acid (SA), 100 µM; jasmonic acid (JA), 50 µM; abscisic acid (ABA), 10 µM; cytokinin (6-BAP), 1 µM. *n*=10 per treatment for each line. Representative images are shown. Scale bar, 0.2 mm. (**b**) A close-up image of 100 nM NAA-treated *PDLP5pro:GUS* root, showing GUS staining over ectopically induced LRPs (brackets). (**c**) Seven-day-old *Arabidopsis* WT seedlings were mock-treated with water or sprayed with the indicated hormones for 9 hours, then roots were excised, frozen, and RNA collected for RT-PCR. Relative band intensity was quantified with Image-J and standardized against ubiquitin. Transcript levels of *IAA19* was included as a control for auxin response. Three biological and two technical repeats were performed. Asterisks, significance determined by student T-test (P<0.01).

Supplementary Figure 4

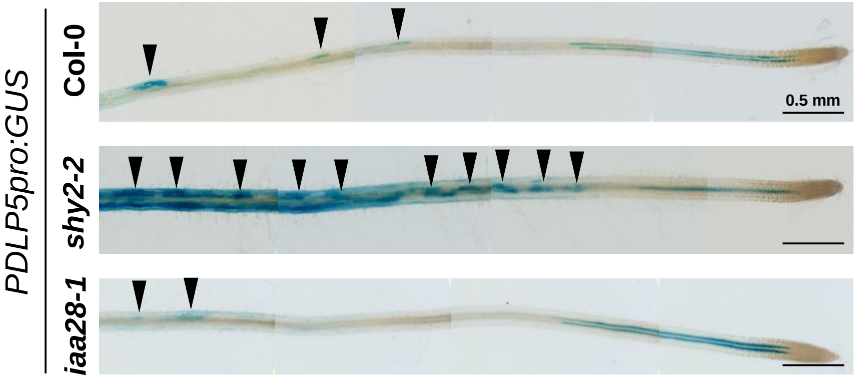

Supplementary Figure 4: *PDLP5pro:GUS* expression patterns in auxin mutants. *PDLP5pro:GUS* expressed in Col-0, *shy2-2* (*Ler*) and *iaa28-1* (*Ws*) mutant backgrounds, showing the changes in staining pattern and intensity. Nine-day old seedlings were GUS-stained for 1 hour. Darts, LR forming or aborted regions.

Supplementary Figure 5

a

```
chr1:26649013-26652012 FORWARD LENGTH=3000; 5’UTR chr1:26652013-26652098 FORWARD LENGTH=86

CAAAAACCAATTGGAAGATACATCTAAATATTACCTTAGATATTACACATTAAATTTTATCAAAAAAATAAAATATATATTACACATTAAACATGAAATAAATAAAAAAGA
AGAAGACTGGATCAGACATTATGCTTATTGTGGTGTCTAATCTAACATACACATGTGTCGTGTTACATTCTTTTAGTTCCCTTTTCAAATAGAAAAACTTTTGTTTAAAGG
GACAGCCAAGTGGGTGAGTTTAAACCTTTTATTCCTCAAAATAATTAGATCCTGACAAATTATGTCTTAATCCACAACTAATCTACTTTCATATATGGCATTGATCAATAT
CTTTATACACTTTCAGGATGTAATGGTCTCTAACTCTCTCTATATATAAGATATGCTCGAATATGTAGCAACAATTGTAAACGAGTATCATATAATCAATAAGACTTTAAA
AAATAATCAGAATCAGATTCTTACTAACCAATTACTGATGCGTAAGAAGAGAAAAGGTGAAAAGATCATAATTATATCTGATCTTGCTATGAACATATTCATGTGAAAATA
GACAGACTTTGATACGAAGAAAACTAAAACAGAGGGAAATTGATCTTGTCAGTTTGTACTATAACTTTTGAATTCTTGCTAGATATTTTACTCTAGCCTCTAAATGAAAAC
GTAAATTTTGAAGGTAAACATAAGACGATCCTAATTATAATTTGTAAGCTAAGATTCTCATATAAATAATATCAGATTAGTTCACTCAAATCTATAATAGGCATAGGGATA
TTGGATGTCTCCACAGATTTTAATCTTGAAAGAGTTCCACAATTTGTCGAAGAAACGTTTTCCTTTTGGAATTGACGAATGAGTCAACTTAATTAGGTTAGTCTAAATACA
AGAAAATTATCGAGTGCCCATTCGAATTTTTTTATTATTTTTCAGGAAATGACCAAAGACTTATTAGAAGTCAAGCACGAAATTACTCACCAAAATCCCTTAATCTCTGCA
TTAGTTTATTTCTCCCTAAATTTATATTTTCTCCATCTAAATGATTACGGATCCACCTGCCAAGGAAAAAAAAAATAACTGTGTTACGAAAAAATTAAAAATCAATACTTGG
TTGGAAAACTAAAACTAAAACCGAACAAAAATCCAAATATGACATTCCTATTCTAATTCAACAATAGAATTAATTATTAGCGTTGGAACAATCTATATATAGATATTAAAG
CTTTGTGTGTGTAAGATAGAAGAGTTCTTAAAAGTCTATTTGTGTGGGTAAGCCATTGATCGTTTAGTGACTAAATGATACAACTTCTAATCCCCTTTATCAAACTTTATC
CAGTCTTTGTTTAGCTGTACTTTTCACTGATATATTTTCTCTTCTTATGTTACGTGAAAATAAAATAATACTATTGAAAAGTAATTTGATTGATCAATTCCACCATGCAAT
TAATTACCAGAACTTTCTGCTTTCAATTATATCTTCTTAATTAATTGGTTATTATTTCAACTTCATAACTGAATAGAATCCCTTATCGACAAAATGATTACTCTTGTATTG
TTACATTATTGTCATTGTTTCCTTAATAATTATCAATGATCTAGTATCATAAATGTTACCTTCTATAGTTAATAGTTCATGTACTAAGTACTAAAACATGTGTAGTATGAA
AAAATACAACATCCATGTTGTAATTAATTTATAGATAGATTCAAGTATACGTACAACAATAAATTCTTTTGCAGCCGTAACGATTTTTTTATTTTTTATTTTTTATTGAAT
ACCAGTAAATTCTGGGCCGAAGCCCGTAATCCCCTCAGGTTCGAAACCACAGCATGTAATATTAGTTTCGTCCCAAGCGTCATTCGAACTGGCGACCTCTAAGATTCGCCT
AAGGTCTTTACTAATTGAGCTACAATGGATAATAAAAGAATCTAACCTTCCAACAAAAAGAAATAGTATTACTAAAGTCAATAAGAACAATCTATAGCCAAAGAAAAGGTC
TATGGGTGTTGTGGTTATAAGAACTAAACAAAAGTCGGTCAAATAACATTATTGTCTTCTCTATTCGTTCACCGGTCCCGTAACTGAACTTTGACGTTGGCAGCCTAGTTA
CTTGATTGATTTCTCATCATACTAATAAAAATAATAAAATGTTGATACAACCCTTACATAAATGTATACAACATCTCGACCAGCACGCCAATATCCCTTTCTATATAATTA
AAATTCATCTTCTAAAATCATTTTCAAGTACCATTGCCTATCCAAACTCTATACGCTATACTACATGTAATATATTAATGACCATAACTCGTTTTCATGACGTATACGTCG
TCATTGCTCGGTATTATTCGTATTGCTTTTTTTTTCTGATGATATAGAAAAACAAAAAAAAAGACCTACTATTGTCAATTTTTATATACACACACATAATGCGAGAATTGC
TTCGTCACTCCTATCATCTGATATAAGAAAAATAAACTAGTTGAATGCAAATTTTCATATCTTTTTGTGTTGATTATATATACATAATACATGAATAAAGAAGTTGGTTTT
TTATTAATTTCGTGGTAAGTCCGCCGATTTGGCCTTTCTATACGCATTCAAACTTCACAATACTATAAGAAATTAACTCCTCAGTTTTATTGCCTTCTTGATAAGTGGCAA
CTGGCAAGTAAGGGTAATTAGTAGTAACGGTAGAGGCTAACGAATTCACACGAGTCACTGTCAAAATAAAAAAATTGAATTCAAATGACATGAGTAAGGATAGATTAACAAA
AAGAAAGTTGTAATGGATAGACGCACATTGCCAAATTCCACATTATAGCTTTAAAAAAAAAAGTCTGAACGAAGAAAGAGTTGCAATTATTTAGTAATTTCAATGATATGG
AACTTGCAAATTGTGGAAATTCTTAAGAACGTCCCCAACCAAAAACCAAAAAAAAAAAAACCAAAAGCATGCAACATGGAACCACATATAAGAGTACCCTTCTTTACGTCA
ATTATCTTTAAAGCAAGTCTTCGCTCTTCAATTTATATTTTACCAAATCATCTTCTCTCTTTCTCTATCTCTCAAAACAAAAAGTAACCATG

GGGACA -2866
TGTCTC -2304
TATA box ATATAAGA-115
Y Patch TCTTCTCT-39
Core TGTC/GACA
```

b

Elements	Position within PDLP5 upstream sequence							
ATG	1							
Transcription Start Site	-86							
Y-Patch	-39							
TATA box	-115							
TGTC (core forward)	-2942	-2920	-2802	-2485	-2265	-1523	-683	-364
GACA (core reverse)	-2811	-2863	-2811	-2531	-1935	-1556	-337	
TGTCTC (forward)	-2304							
GAGACA (reverse)								

Supplementary Figure 5: The *PDLP5* promoter contains auxin-regulated elements. (a) Upstream sequence of *PDLP5* showing promoter and auxin-regulated elements. **(b)** Position of promoter and auxin-regulated elements.

Supplementary Figure 6

a

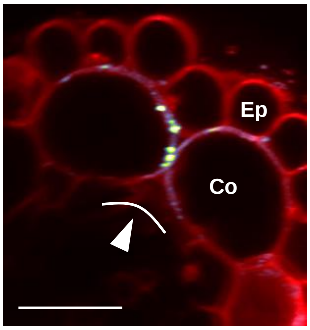

PDLP5pro:PDLP5-GFP
in *pdlp5-1*

b

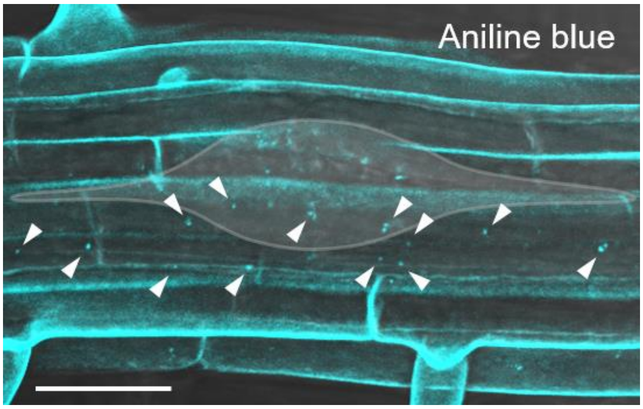

Supplementary Figure 6: PDLP5-GFP induction and callose accumulation in overlying cells. (**a**) A representative confocal image of *PDLP5pro:PDLP5-GFP* expression pattern in the *pdlp5-1* mutants. Arc with dart, position of LRP; scale bars, 25 µm. (**b**) Six-day-old WT seedlings were stained for 30 minutes in aniline blue stain to reveal callose accumulation at PD (darts) in LRP-overlying cells. The grey outline shows the position of the LRP. Scale bar, 50 µm.

Supplementary Figure 7

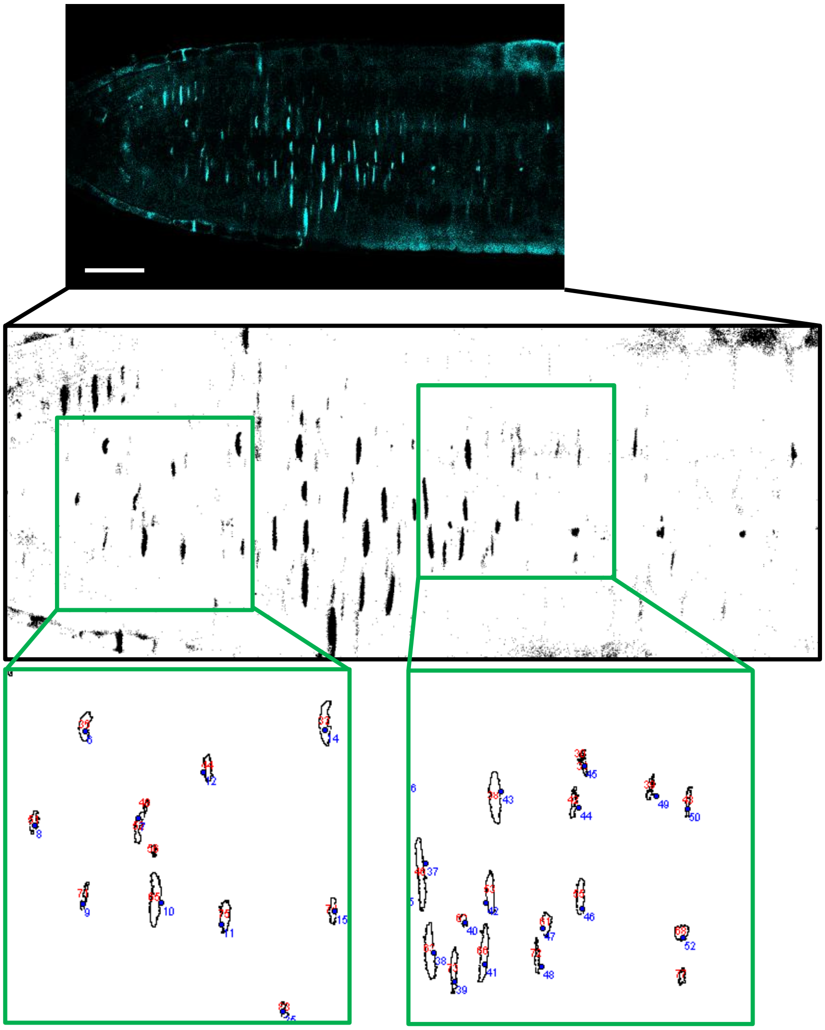

Supplementary Figure 7: Aniline blue-stained callose quantification. Six-day-old *pER8:PDLP5* seedlings were stained with aniline blue for 5 minutes after 24 hours of 10 µM estradiol or mock treatment. Images were taken at the root tips. Quantification of fluorescence intensity was done using the Threshold tool in ImageJ software to eliminate background staining, and the number of cell-cell junctions with aniline blue-stained callose within the root tips were counted per seedling. Scale bars, 25 µm.

Supplementary Figure 8

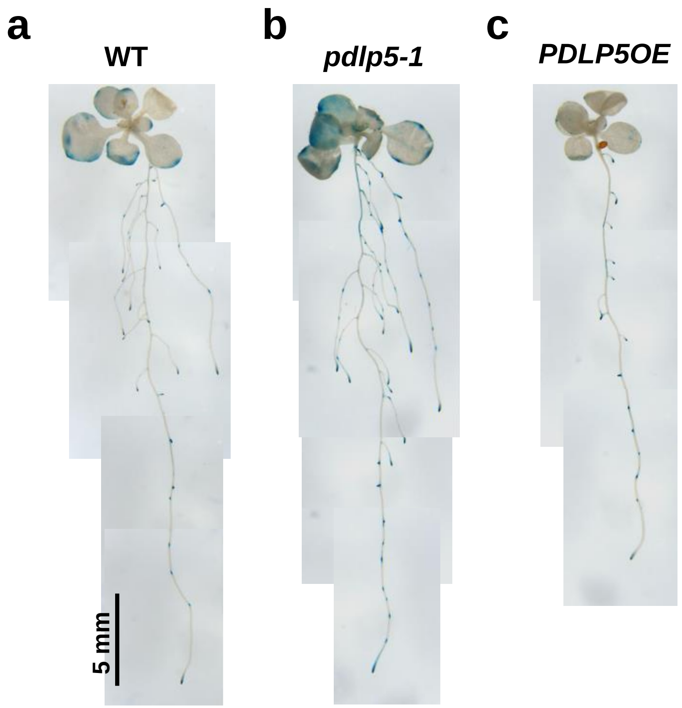

Supplementary Figure 8: Root branching patterns in *pdlp5-1* and *PDLP5OE*. Images of GUS-stained 11-day-old seedlings expressing *DR5:GUS* in WT Col-0 (**a**), *pdlp5-1* (**b**), and *PDLP5OE* (**c**) backgrounds. Representative transgenic lines are shown. Scale bar in (**a**), common to (**b**) and (**c**).

Supplementary Figure 9

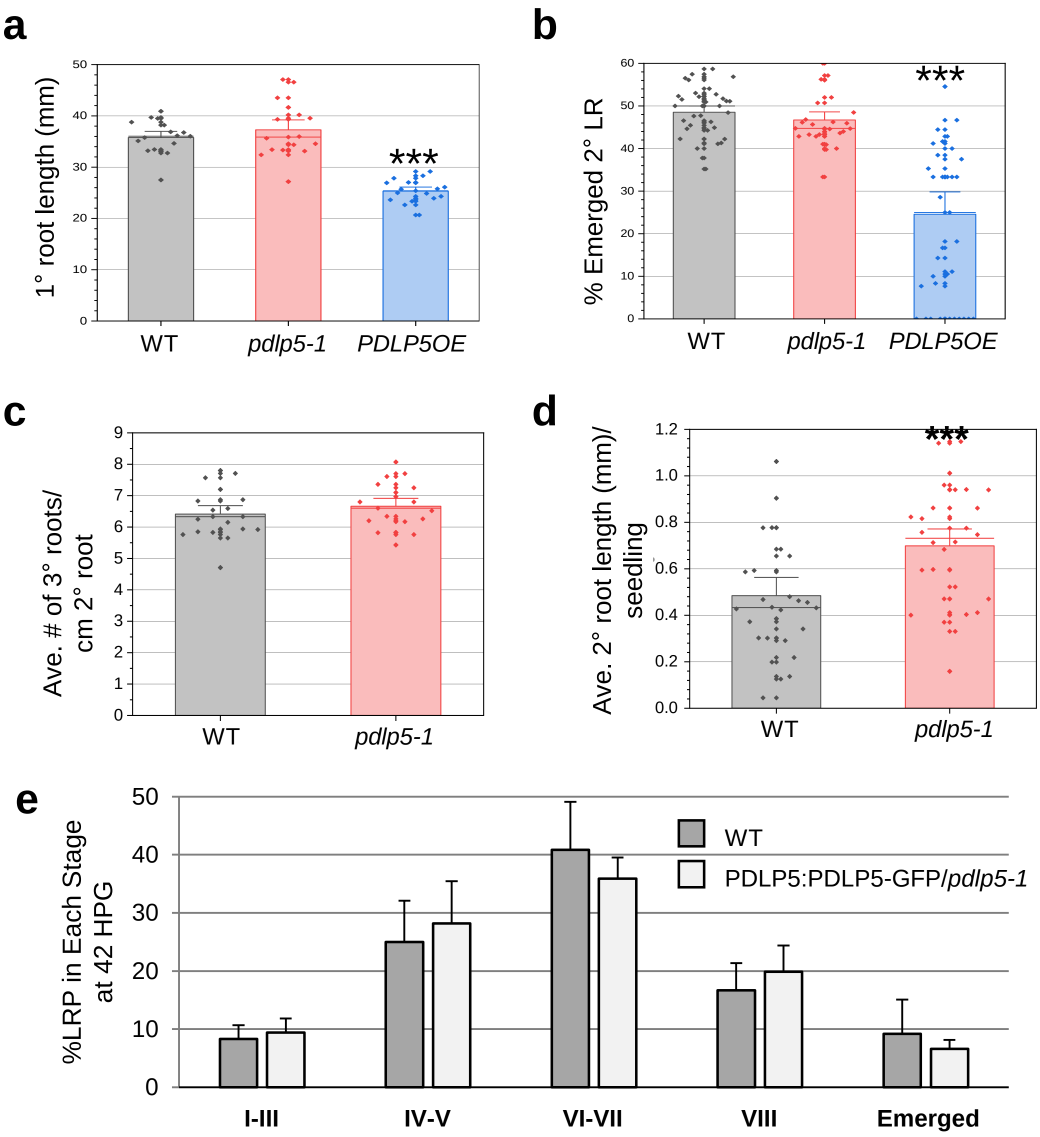

Supplementary Figure 9: Root number and length measurements of *pdlp5-1 and PDLP5OE*. (**a**) Total primary root length is similar in WT and *pdlp5-1*, but reduced in *PDLP5OE*. (**b**) The percent of emerged secondary roots per seedling was similar in WT and *pdlp5-1*, but reduced in *PDLP5OE*. (**c**) The tertiary root density is similar between WT and *pdlp5-1* backgrounds. (**d**) Average secondary root length per seedling was higher in *pdlp5-1* than in WT. (**a**) and (**b**), seedlings 10 dpg, *n*=30 per line; (**c**) and (**d**), seedlings 7 dpg, *n*>20 per line. (**e**) *PDLP5pro:PDLP5-GFP* rescues the *pdlp5-1* LRP emergence phenotype. *PDLP5:PDLP5-GFP* in *pdlp5-1* *n*=31; WT *n*=35. Asterisks, significance determined by student T-test (P<0.001).

Supplementary Figure 10

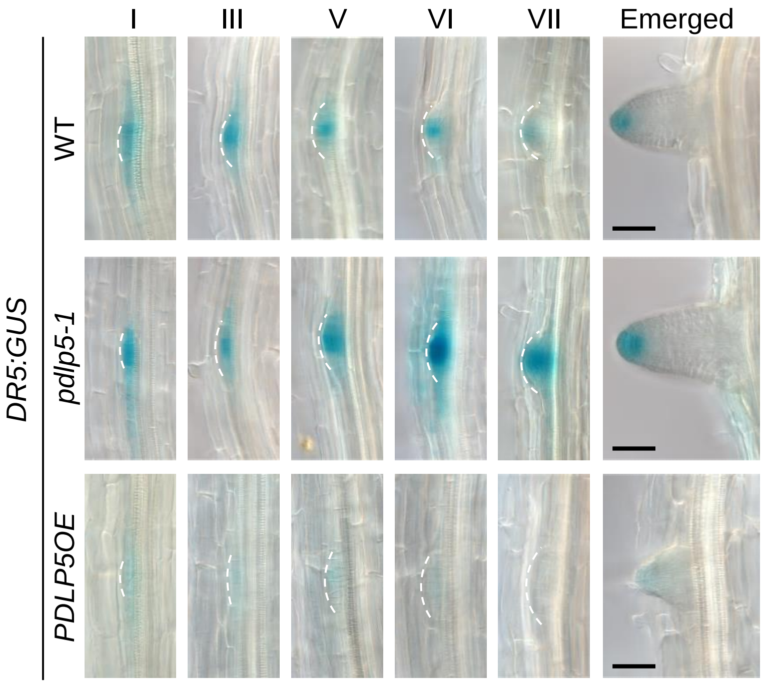

Supplementary Figure 10: *DR5:GUS* intensities at the LR tips. Seven-day-old *Arabidopsis* seedlings expressing *DR5:GUS* were GUS-stained for 25 minutes and observed under the microscope after clearing in 85% lactic acid for 2 hrs. Occasionally, increased *DR5:GUS* staining within the LRP zone in *pdlp5-1* at mid-to-late emergence stages (V-VII in the figure) was observed compared to WT. Commonly, there was an overall decrease in DR5:GUS staining within LRP in the *PDLP5OE* background; this could be due to repressed auxin signaling via negative feedback by PDLP5, or due to side-effects of the SA hyper-accumulation within this background. LRPs outlined in dashed lines; scale bars 50 µm, common to all panels.

Supplementary Figure 11

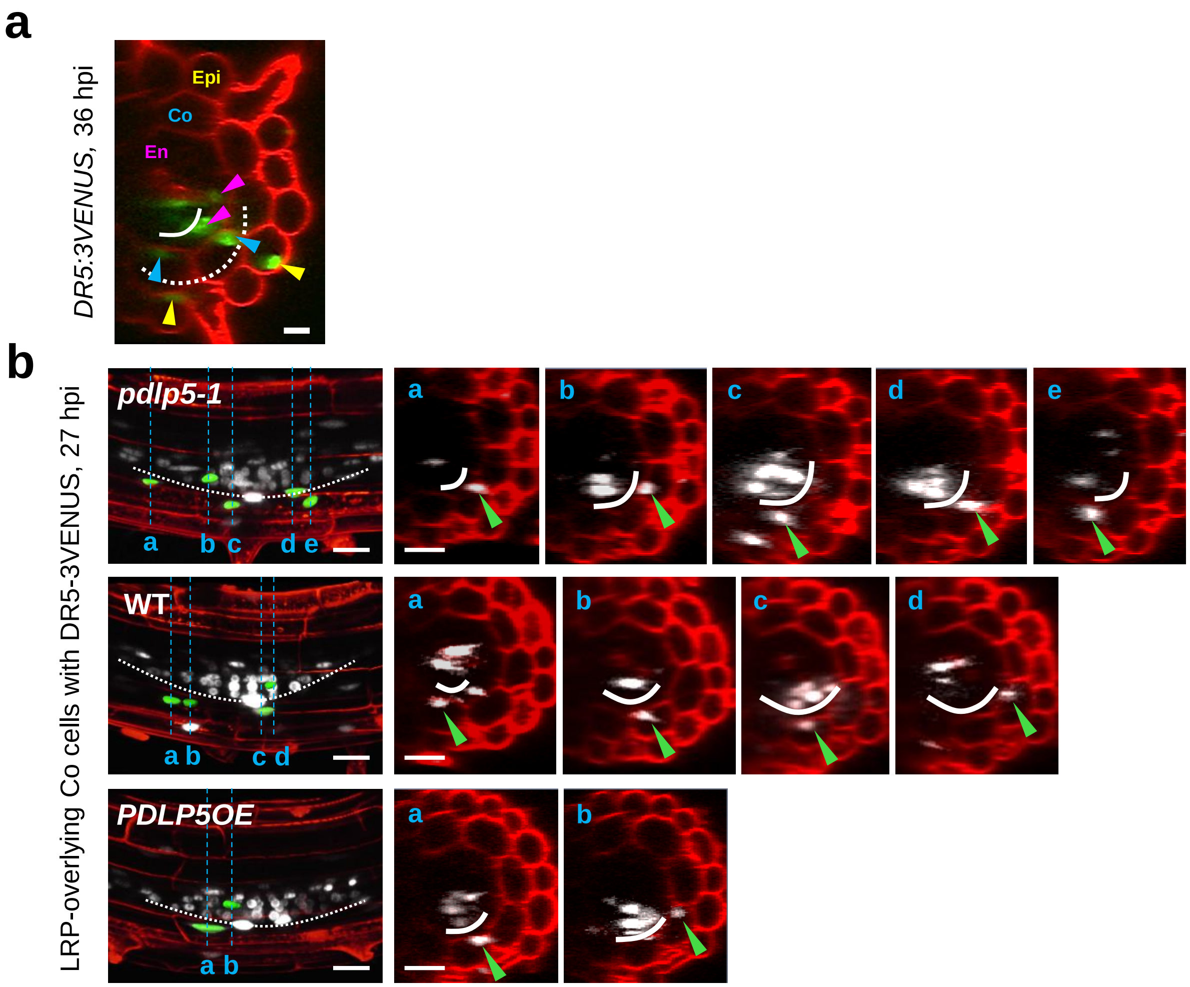

Supplementary Figure 11: *DR5:3VENUS* expression in LRP-overlying cortical cells. (**a**), Representative image of a PI-stained 36 hpg root bend LRP in *DR5:3VENUS*, shown as a single latitudinal cross-section from a confocal z-stack image. The solid white curve outlines the portion of the LRP visible within this single cross-section; the dotted white curve represents the maximum size of the LRP from the whole confocal image. Arrowheads indicate *DR5:3VENUS*-expressing LRP-overlying cells (green nuclei) visible within this single image: yellow, epidermis; blue, cortex; pink, endodermis. Scale bar, 20 µm. (**b**), Leftmost panels are 2D maximum intensity projections of 115.4 µm thick confocal volumes, showing representative images of *DR5:3VENUS* signal in the nuclei of emerging LRP and surrounding cells at the root bend in each background; LRP-overlying cortical nuclei expressing *DR5:3VENUS* are pseudo-colored green, while all other *DR5:3VENUS*-expressing nuclei are white. The WT panel has a white arc representing the overall shape of each LRP. The vertical blue dotted lines in the leftmost panels represent cross-sectional planes shown in the panels to the right; each cross-section is through the nucleus of an LRP-overlying cortical cell expressing *DR5:3VENUS* (marked by green arrowheads in the cross-sectional planes to the right). Scale bars, 25 µm.

Supplementary Figure 12

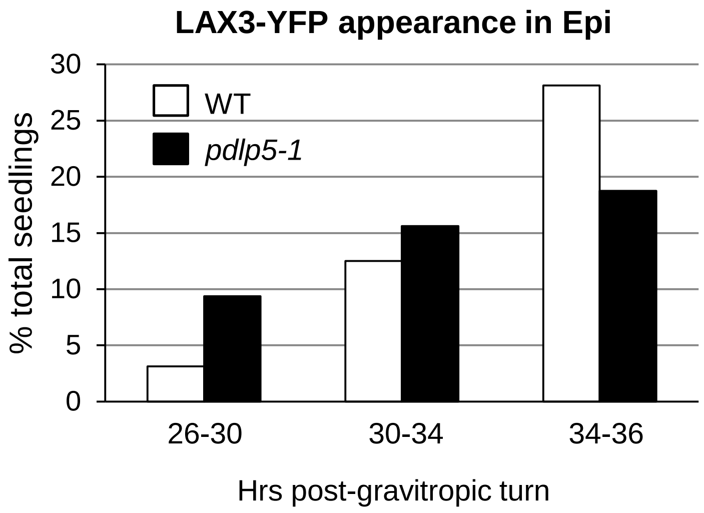

Supplementary Figure 12: LAX3pro:LAX3-YFP induction timing in overlying epidermis. Timing of LAX3-YFP appearance in the Epi layer of the above seedlings observed between 26-36 hpi. *n*=32.

Supplementary Figure 13

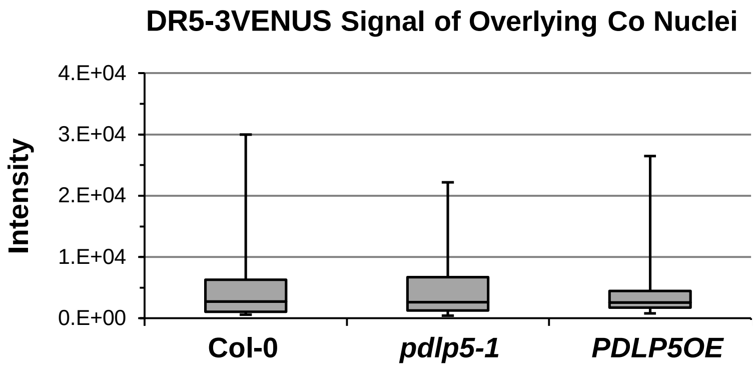

Supplemental Figure 13: A box plot analysis of the fluorescence intensity of DR5-3VENUS signal in LRP-overlying cortical nuclei in WT (Col-0), *pdlp5-1*, and *PDLP5OE* backgrounds. The fluorescence intensity data were analyzed using ImageJ software. The median section through each nucleus in the 3D z-stack was imaged separately and converted to a 2D 16-bit grayscale, then the Threshold function was used to select the nuclear area of the converted image and measure its mean intensity. The DR5-3VENUS fluorescence intensities of all LRP-overlying Co nuclei varied widely. n>20 nuclei for each background.

Supplementary Figure 14

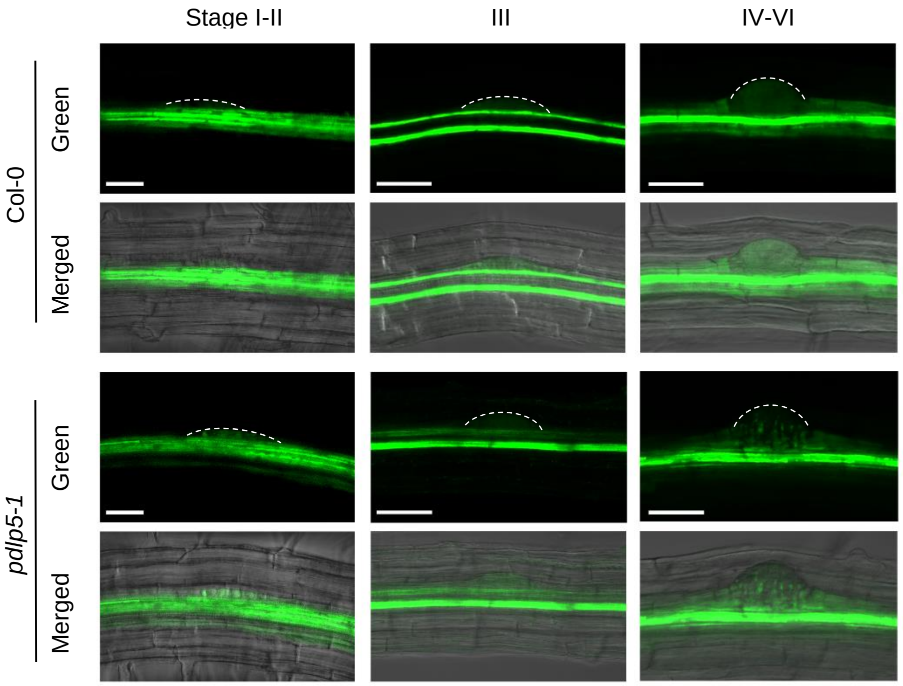

Supplementary Figure 14: CF unloading into emerging LRPs. Confocal images showing CF accumulation in nascent and emerging LRPs. Col-0 and *pdlp5-1* seedlings grown on MS agar plates for 10-14 days were phloem loaded with 1 mM CFDA as 1 µL droplets on two leaves that were gently crimped with a fine-tip tweezer. Confocal images were taken between 30-75 min post phloem loading, using 480-nm excitation line of an argon laser and Apochromat 25x/0.8 Imm Korr DIC M27 objective on a Zeiss LSM880 multiphoton confocal microscope. A maximum projection of the CF excitation was generated (green) and merged with a signal frame of the transmitted light channel. More than 40 seedlings were examined for each genotype. Scale bars, 50 µm.

Supplementary Table 1. Quantification of seedlings expressing LAX3pro:LAX3-YFP in overlaying Co cells at 22 hours post-gravitropic induction.

**Repeats**	**WT**	***pdlp5-1***	***PDLP5OE***	***pdlp5-1*:WT**	***PDLP5OE*:WT**
Set 1	6/23 (26%)	9/20 (45%)	0/21 (0%)	1.73	0
Set 2	5/43 (12%)	10/40 (25%)	0/23 (0%)	2.08	0
Set 3	7/34 (21%)	13/37 (35%)	0/24 (0%)	1.67	0
Total # of seedlings	100	97	68		
Average	18%	33%	0%	1.83	0

Supplementary Table 2. *LAX3pro:LAX3:YFP* cortical signal in *PDLP5OE* at 24 and 36 hours post-gravitropic induction.

**Repeats**	**WT (24 hpg)**	***PDLP5OE* (24 hpg)**	***PDLP5OE* / WT (24 hpg)**	***PDLP5OE* / WT (36 hpg)**
Set 1	11/31 (36%)	4/27 (15%)	0.42	1
Set 2	22/30 (73%)	15/30 (50%)	0.68	1
Total # of seedlings	61	57		
Average	55%	33%	0.55	1

Supplementary Table 3. Genetic crosses used in this study.

Maternal line	Paternal line	Cross used in study
PDLP5pro:GUS	shy2-2	PDLP5pro:GUS x shy2-2 (F2 showing root phenotype and GUS staining)
PDLP5pro:GUS	iaa28-1	PDLP5pro:GUS x iaa28-1 (F2 showing root phenotype and GUS staining)
LAX3pro:LAX3-YFP	pdlp5-1	LAX3pro:LAX3-YFP x pdlp5-1 (F3 homozygous for pdlp5-1, segregating LAX3-YFP)
LAX3pro:LAX3-YFP	35S:PDLP5	LAX3pro:LAX3-YFP x 35S:PDLP5 (F3 homozygous for 35S:PDLP5, segregating LAX3-YFP)
CASP1pro:GFP	pER8:PDLP5	CASP1pro:GFP x pER8:PDLP5 (F1 and F2 to observe GFP movement before and after inducing PDLP5)
PDLP5pro:PDLP5-GFP	pdlp5-1	PDLP5pro:PDLP5-GFP x pdlp5-1 (F3 homozygous for pdlp5-1, complementation studies)
DR5:GUS	pdlp5-1	DR5:GUS x pdlp5-1 (F3 double homozygous, for root phenotyping)
DR5:GUS	35S:PDLP5	DR5:GUS x 35S:PDLP5 (F3 double homozygous, for root phenotyping)
DR5:3VENUS	pdlp5-1	DR5:3VENUS x pdlp5-1 (F3 homozygous)
DR5:3VENUS	35S:PDLP5	DR5:3VENUS x 35S:PDLP5 (F3 homozygous)

Supplementary Table 4. PCR Primers used in this study.

**Primer name**	**Sequence (5'→3')**	**Purpose**
Lpr852	TTTGCATAGACGAAAAACATGG	Genotyping for *pdlp5-1* allele
Lpr853	TGGATCTTACAGGACAGGTGG	
SAIL LB1	CCTTTTCAGAAATGGATAAATAGCCTTGCTTCC	
Lpr136	GGAAGACCATAACCCTTGAGGTTG	RT-PCR for UBQ5 transcript levels
Lpr151	TCTTAGCACCACCACGGAGA	
Lpr665	ACAGCCGATGGAATTCAACG	RT-PCR for PDLP5 transcript levels
Lpr723	CTTCTCTCCTTCATGACCAAAGT	
Lpr1269	GAGCATGGATGGTGTGCCTTAT	RT-PCR for IAA19 transcript levels
Lpr1270	TTCGCAGTTGTCACCATCTTTC	
KH318	GTTCACTCAAATCTATAATAGGCATAGG	PDLP5 promoter -2341 to -2260
KH319	CGACAAATTGTGGAACTCTTTCA	
KH320	GGTAGAGGCTAACGAATTCACA	PDLP5 promoter -394 to -285
KH321	GTGCGTCTATCCATTACAACTTTC	
KH69	TGCATTGGTACACAGGTGAGGGAA	TUB3 (At5g62700) control primer pair: exon 3 (+1756 to 1863)
KH70	AGCCGTTGCATCTTGGTATTGCTG	
KH322	CACAATGTTTGGCGGGATTGGTGA	Actin12 (At3g46520) control primer pair: exon 3 (+1095 to 1199)
KH323	TGTACTTCCTTTCCGGTGGAGCAA	
Lpr1303	GAAGATCTGCGGCCGCAAACAAAACATATCTCAATTTCATGAC	Clone PDLP5 promoter with NotI/AscI R.E. sites
Lpr1304	CCGCTCGAGGCGCGCCGGTTACTTTTTGTTTTGAGAGATAGAG	
Lpr1428	GAAGATCTGCGGCCGCTTAATCTGCATAAAAGTGAGTATGAG	Clone CASP1 promoter with NotI/AscI R.E. sites
Lpr1429	CCGCTCGAGGCGCTTTCTCTTGCAATTGGGGTTTAAAAG	
Lpr1336	GACTCGAGAATGAAGACTAATCTTTTTCTCTTTCTCATCTTTTCACTTCT	Clone ER-YFP with HDEL sequence and XhoI/XbaI R.E. sites (PCR#1, Lpr1337 and 1338; PCR#2, Lpr1336 and 1338)
Lrp1337	CTCATCTTTTCACTTCTCCTATCATTATCCTCGGCCGTGAGCAAGGGCGAGGAGCT	
Lpr1338	AATCTAGATTAAAGCTCATCATGCTTGTATAGCTCGTCCATGCCG	
